# Root exudates in controlled environment agriculture: composition, function, and future directions

**DOI:** 10.3389/fpls.2025.1567707

**Published:** 2025-04-28

**Authors:** Deniz Camli-Saunders, Camilo Villouta

**Affiliations:** Controlled Environment Agriculture Lab, Department of Plant Sciences and Entomology, University of Rhode Island, Kingston, RI, United States

**Keywords:** root exudates, rhizosphere interactions, allelopathy, microbiota interactions, hydroponics, controlled environment agriculture (CEA), soil-based agriculture

## Abstract

Two decades of research has revealed an intricate network of root exudates in plants, which they use to interact with and mediate their surrounding environment, the rhizosphere. Prior research has been conducted mainly on model plants such as Arabidopsis or staple monoculture crops like maize, soybean, and rice, revealing crucial roles in plant growth, microbiota interaction, nutrient acquisition, and bioremediation. However, similar research has only begun to be conducted in Controlled Environment Agriculture (CEA) systems, leaving a considerable knowledge gap in the mechanisms, impacts, and uses of exudates in CEA. Exhaustive literature searches revealed less than two dozen articles with direct implications in CEA vegetable crop exudates. This review synthesizes the existing literature to examine the composition, functions, and influences of vegetable root exudates within CEA systems. The first section explores key compounds —including amino and organic acids, and sugars— along with mechanistic processes, and microbial interactions. The second section compares root exudates in soil-based versus hydroponic CEA systems based upon differences in substrate, (a)biotic stressors, microorganisms, and nutrient availability. By contrasting existing literature on both CEA soil-based and hydroponic systems, the section examines likely differences in exudate composition, mechanisms, and functions. The final section presents case studies from both hydroponic and soil based systems, highlighting how root exudates contribute to environmental stress mitigation, allelopathy, disease response, bio/phytoremediation, and pest control. It reveals major avenues for the use of exudates in CEA systems worldwide. Lastly, we ponder the future avenues of exploration for CEA root exudates, proposing the creation of a database for usage in smaller or organic farms and in urban agriculture settings. In conjunction, we emphasize the need for further investigation into the potential of exogenous applications of exudate-like compounds to positively impact yield, disease resistance, soil restoration, and land reclamation, especially in the context of climate change.

## 1 Introduction

Over the last twenty years, much research has been done in the areas of root exudates. Plants, as sessile organisms, utilize root exudates to respond to and mediate their surrounding soil environment known as the rhizosphere. Up to 21% of carbon allocated to the roots are exuded into the surrounding environment (Upadhyay et al., 2022). Prior research has demonstrated that the allocation of root carbon is highly intertwined with the surrounding water and nutrient cycles (Canarini et al., 2019). Root Exudation is mostly a passive process, but active mechanisms have been recently linked to exudate secretion (Badri and Vivanco, 2009). Efforts to quantify and qualify exudates have been challenging due to the destruction of roots during exudate collection and contamination by other organisms in the rhizosphere. Thus far, the organic and inorganic compounds secreted by plant roots into the surrounding rhizosphere have primarily been studied in so-called “model” plants. Plants such as *arabidopsis*, along with crops crucial to monoculture systems including maize (Hu et al., 2018), soybean (Sugiyama, 2019), and rice (Matsushima et al., 2021) have been the central focus of studies worldwide. This has left a gap in the literature regarding crops in Controlled Environment Agriculture systems. Controlled Environment Agriculture (CEA) is a fast-growing industry which saw at least $900 million in sales in the United States alone in 2022 (USDA National Agricultural Statistics Service, 2022). It has been hailed as a keystone to sustainable production in a rapidly industrializing world. The combination of quick production time and high planting density has allowed it to flourish among the indoor fruit and vegetable production industry. The world population is projected to continue to grow at a rapid pace. Furthermore as several major countries in the global south industrialize, global CEA production is projected to continue to grow. A global goal of efficient resource utilization can be achieved through expanding our database of root exudates, their functions, pathways, and impacts on plants and production systems. When CEA crops have been studied, their focus has been in combating disease and response to stress, rather than strictly qualitative or quantitative analysis of the mechanisms at play (Mavrodi et al., 2021; Moncada et al., 2020). This lack of focus on qualitative analysis has left the scientific community without a database of root exudates that would otherwise be found for crops such as maize, soybean, and rice. Controlled Environment Systems ranging from simple greenhouses to highly advanced, closed loop, nutrient film technique hydroponic systems have yet to be studied with regard to the mechanisms and role of exudates in these respective systems. There is evidence from traditional soil agriculture which demonstrates a key role for root exudates to aid in crop production (Hnini and Aurag, 2024; Upadhyay et al., 2022; Mavrodi et al., 2021). A role may exist in CEA systems for exudates, particularly with regard to allelopathy, yield increase, and carbon fixation (Eze and Amuji, 2024; Zhou et al., 2023). In synthesizing this review, we have attempted to compile a list of the common root exudates secreted by CEA crops, along with their common functions in the plant and surrounding rhizosphere. The first section of this review describes the amino acids, organic acids, and sugars commonly exuded by CEA crops. It explores their functions, and possible relationships that exudates can have with microbiota. The second section focuses on case studies with regards to specific crops. It encompasses water stress conditions, substrate relationships, and allelopathy– all situations which provide novel benefits to CEA crop production. The review concludes with an overview of relationships and major findings, and ponders the future directions for root exudate research in CEA crops.

## 2 Compounds, functions, and microbial interactions

Root exudates can be broadly separated into two categories: Low Molecular Weight Compounds (LMWC) and High Molecular Weight Compounds (HMWC) (Vives-Peris et al., 2020; Upadhyay et al., 2022). The former is made up of ions including H^+^, various inorganic acids, and all twenty amino acids. Various carbohydrates and sugars including Glucose, Fructose, Galactose, and Sucrose can be present as well (Mavrodi et al., 2021). Phenocarboxylic acids, and various organic acids including acetic acid, citric acid, caffeic acid, and malic acid round out the Low Molecular Weight Compounds. HMWC consist of larger polysaccharides including cellulose and starch, proteins, and various phenolics including tannins (Badri and Vivanco, 2009). Vegetable and fruit crop exudates in CEA systems primarily consist of three compounds. These are amino acids, organic acids, and sugars (Canarini et al., 2019; Vives-Peris et al., 2020; Vančura and Hovadík, 1965).

### 2.1 Amino acids

While roots of vegetable and fruit crops can and do exude all twenty amino acids, the most common are found to be α-Alanine, β-alanine, arginine, asparagine, cystine/cysteine, glutamine, glycine, histidine, lysine, methionine, phenylalanine, proline, and serine (Vančura and Hovadík, 1965; Trovato et al., 2021). Each serves a function when exuded from the roots, as well as in general plant metabolism. Amino acids are categorized as “Low Molecular Weight Compounds” (Canarini et al., 2019). They can be categorized into subgroups based upon the location of structural functional groups. These subgroups include “alpha, beta, and gamma” designations (Trovato et al., 2021). Roots exudates are dominated by the “alpha” amino acid subgroup. These alpha amino acids are made up of a central carbon atom that is bonded to the amine group as well as a carboxylic group (Trovato et al., 2021). Beta and Gamma subgroups have a differing structure and make up various peptides and compounds such as Butyric Acid (gamma) or β-alanines. While nearly all of the aforementioned amino acids have a role in protein or enzyme synthesis, several of them have other specialized roles as well. α-Alanine’s aid in the process of photosynthesis, while β-alanines are built up in higher concentrations in response to biotic and abiotic stressors (Parthasarathy et al., 2019).

**Figure 1:**
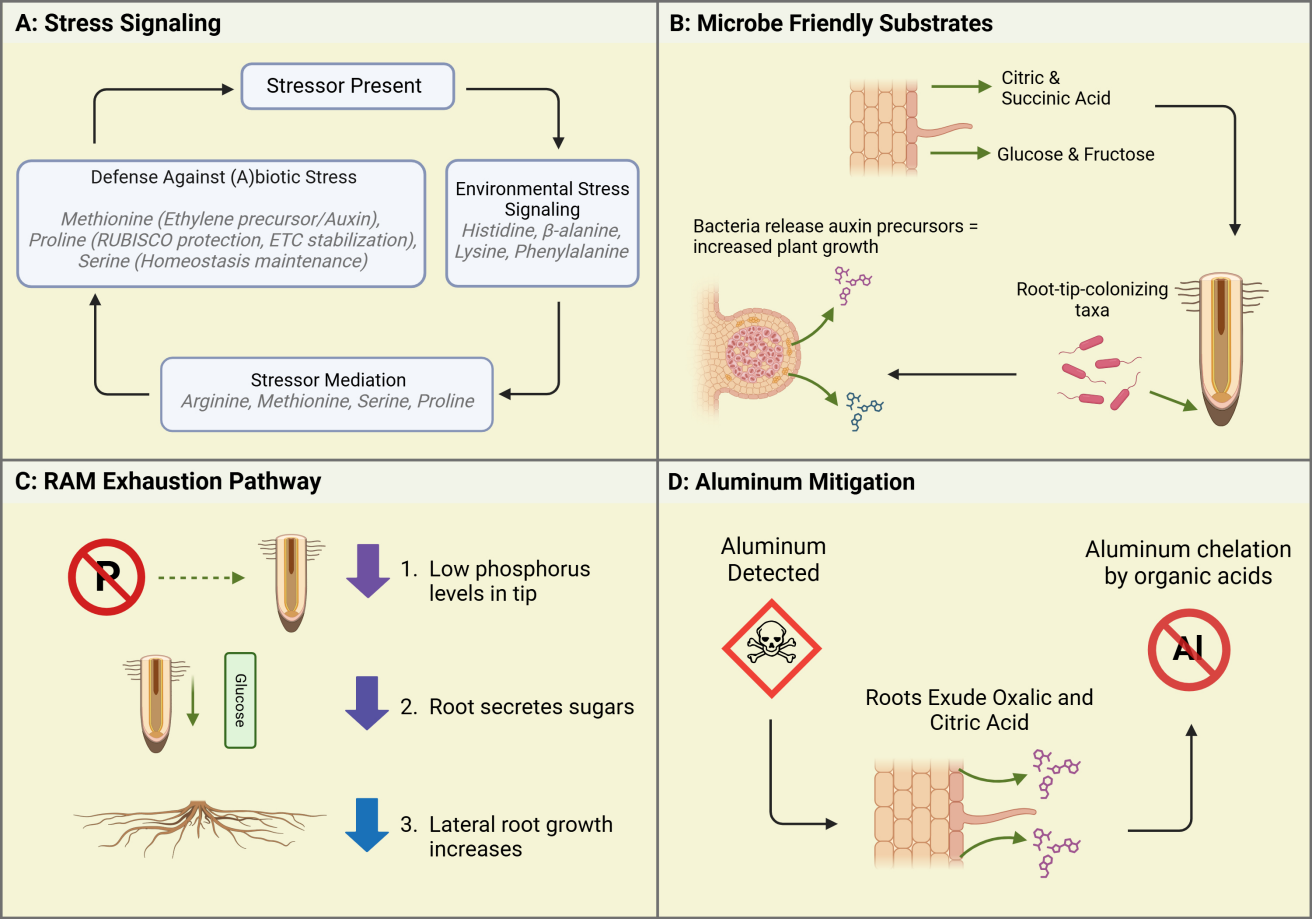
Case studies illustrating root exudate functions in response to environmental conditions within Controlled Environment Agriculture (CEA). **(A)** Stress Signaling: When a stressor is present, plants initiate environmental stress signaling through the exudation of specific amino acids. In response, stressor mediation occurs via compounds like arginine, methionine, serine, and proline, which help regulate plant stress responses. Finally, plants deploy defense mechanisms against abiotic and biotic stressors by exuding compounds such as methionine, proline, and serine, contributing to overall stress resilience. **(B)** Microbe-Friendly Substrates: Root exudates, including organic acids and sugars, facilitate beneficial microbial colonization. Microbes respond by releasing auxin precursors, enhancing plant growth and recruiting root-tip-associated microbial taxa. This process is particularly relevant in hydroponic and soilless systems, where microbial recruitment differs from traditional soil environments. **(C)** RAM Exhaustion Pathway: Under phosphorus deficiency, plants alter root growth dynamics by exuding sugars at the root tip. This signaling mechanism suppresses primary root elongation while stimulating lateral root proliferation, improving phosphorus acquisition efficiency under nutrient-limited conditions. **(D)** Aluminum Mitigation: When aluminum toxicity is detected, roots exude organic acids to chelate aluminum ions. This detoxification mechanism prevents aluminum from interfering with root growth, highlighting an essential plant survival strategy in acidic or contaminated environments. Created in BioRender. Villouta, C. (2025) https://BioRender.com/r75b815.

Biotic stressors include predators and microbial attacks. Abiotic stressors include temperature fluctuations, toxic metal accumulation, anoxic conditions, and drought conditions (Vescio et al., 2022). Arginine is involved in nitrogen preservation and recycling, and helps the plant adapt to abiotic stressors (Yang and Gao, 2007). It accumulates in the roots during temperature drops. Asparagine is a non-essential amino acid synthesized in the reproductive tissue of plants. It serves to facilitate nitrogen transport in developing tissues (Sieciechowicz et al., 1988). Cysteine is synthesized to break down sulfur found in the soil (Romero et al., 2014). Sulfur is an important component of enzymatic activity and nitrogen metabolism in plant growth (Xiao et al., 2023). Glycine and glutamine have a role in iron chelation and chlorophyll synthesis respectively (Kan et al., 2015). As well, nitrates and ammonium can be converted into glutamines to make them available for plant use, increasing photosynthetic capacity (Kan et al., 2015). Histidine, lysine, and phenylalanine have roles throughout the plant in signaling responses to environmental stressors (See **Figure 1A**) (Lopez and Mohiuddin, 2023). Methionine acts as a precursor to ethylene, the hormone which cross-communicates with Auxin to mitigate plant growth and regulate the stress response (Trovato et al., 2021). Proline aids in recovery from plant stress by protecting RUBISCO and stabilizing the electron transport chain (Hayat et al., 2012). Serine is a key amino acid in maintaining homeostasis. It helps in signaling against environmental and abiotic stressors and threats (Ros et al., 2014). More recently, it has been found that serine also aids in the synthesis of other amino acids and phospholipids, marking its utility throughout the entire plant (Ros et al., 2014). In summary, amino acids most commonly exuded by vegetable and fruit crops in CEA systems can be further divided into three main functions (see **Table 1**). First, there are those that signal against environmental stressors. Second, those compounds which help to mediate or alleviate the environmental stressors. Third, are the compounds which facilitate nutrient uptake or transport from the rhizosphere and surrounding soil.

**Table 1:** Amino Acid Exudates in CEA Crops.

Amino Acid	Amino Acid Group	Function	Citation
Histidine	Group IV: Basic	Env Stress Signaling	Lopez and Mohiuddin, 2023
β-alanine	Group I: Nonpolar	Env Stress Signaling	Parthasarathy et al., 2019
Lysine	Group IV: Basic	Env Stress Signaling	Lopez and Mohiuddin, 2023
Phenylalanine	Group I: Nonpolar	Env Stress Signaling	Lopez and Mohiuddin, 2023
Arginine	Group IV: Basic	Stress Mediation	Yang and Gao, 2007
Methionine	Group I: Nonpolar	Stress Mediation	Trovato et al., 2021
Serine	Group II: Polar, Uncharged	Stress Mediation	Ros et al., 2014
Proline	Group I: Nonpolar	Stress Mediation	Hayat et al., 2012
Asparagine	Group II: Polar, Uncharged	Nutrient Uptake Facilitators	Sieciechowicz et al., 1988
Cysteine	Group II: Polar, Uncharged	Nutrient Uptake Facilitators	Romero et al., 2014
Glycine/Glutamine	Group I: Nonpolar	Nutrient Uptake Facilitators	Kan et al., 2015
α-Alanine	Group I: Nonpolar	Nutrient Uptake Facilitators	Parthasarathy et al., 2019

There are those amino acids which are less commonly exuded but whose functions should still be described here. These include tyrosine, valine, and citrulline (Mavrodi et al., 2021). Plants utilize tyrosine in a myriad of ways though most commonly as a precursor to other biological compounds and metabolites. Notably, tyrosine is a precursor to making 3,4-Dihydroxyphenylalinine (DOPA) (Schenck and Maeda, 2018). DOPA functions as an allelochemical when exuded by plants, and can therefore inhibit the growth of plants in close proximity. Valine is another less commonly exuded amino acid which helps to form an ideal environment for beneficial plant microbes. Specifically, it aids in chemotaxis, and stimulates biofilm formation– an important aspect for the rhizosphere (Chen et al., 2021). Citrulline is a non-protein amino ac, which has specific importance during abiotic stressors. In particular, it aids in maintaining cellular osmolarity in vegetative tissues of various vegetable crops. While the specific pathway has yet to be fully mapped out, novel studies indicate that citrulline acts as a ROS scavenger in drought stress, thereby protecting tissues from oxidative damage (Song et al., 2020). Though they may not be as commonly exuded, such amino acids still have relevant roles in protecting and aiding plants as exudates.

### 2.2 Organic acids

Vegetable and fruit crops in CEA systems primarily exude four organic acids into the surrounding rhizosphere. These are Citric Acid, Tartaric Acid, Lactic Acid, and Aspartic Acid (Kasukawa and Miyazawa, 2020). Citric acid secretions appear to contribute to increases in dissolved organic carbon content. Furthermore, they are a key component of the sulfur and iron redox reaction cycles occurring in the soil. Increased exudation of Citric acid is significantly linked to increases in iron and sulfide concentrations—both of which are necessary for photosynthetic processes (Xiao et al., 2023). Over time, Citric acid exudation can also lower soil pH (Xiao et al., 2023). Tartaric acid, which occurs in most fruits, works in conjunction with Citric acid. It can help with phytoextraction (Khan et al., 2022)—that is the removal of contaminants in the ground where they are then concentrated in shoot tissues. Research by Khan et al. (2022) showed that in conjunction with Citric acid, Tartaric acid enhanced the removal of lead from the environment in turnip crops. Tartaric acid soil amendments have yet to be studied in hydroponic fruit and vegetable crops, and other CEA systems. Lactic acid secretion appears to attract an array of beneficial microbes known as Lactic acid bacteria, or LAB (Murindangabo et al., 2023). These gram positive, facultative aerobes are extremely common across a vast array of environments including the human microbiome as well. In plants, they have several benefits. First, studies indicate that they can serve as a biocontrol agent. This biocontrol translates across bacteria, fungi, insects, and predators (Murindangabo et al., 2023). Increased LAB concentrations led to decreased pest populations through a release of secondary metabolites. Furthermore, these LAB can degrade various polysaccharides, and increase the availability of other nutrients. Plants grown in conjunction with LAB show significant increases in biomass as well as root and shoot length (Lamont et al., 2017). With these increases in bioavailability, LAB has a stabilizing effect on soil structure, and increases water holding capacity (Lamont et al., 2017). Aspartic acid supports a number of functions which are critical to plant survival. Most notably in general metabolism, it serves as a basic amino acid that along with nitrogen and carbon, serves as a backbone of dozens of metabolic processes (Lei et al., 2022). As part of the Krebs cycle, aspartic acid helps to synthesize several of the organic acids in the process. Aspartic acid also aids in the creation of other amino acids, nucleotides, and sugars. As part of the rhizosphere when exuded by plant roots, it helps to assimilate inorganic nitrogen into plant available forms. Under environmental stress, plants can secrete or build up their Aspartic acid content to mitigate drought, salt, heavy metal or even heat (Lei et al., 2022). Aspartic acid remains a critical component of the plant life cycle.

Less commonly exuded, but still present organic acids include fumaric and azelaic acid which play important roles in microbe recruitment and defense priming respectively (Mavrodi et al., 2021). Fumaric acid is most well known as a key intermediate in the citric acid cycle but as an exudate, fumaric acid acts as a source of carbon source for the dozens of species of microbes and microorganisms inhabiting the rhizosphere (Eisenhaur et al., 2017). This carbon can also act as a signal to aid the recruitment of said microorganisms and help create an ideal environment for their growth. The exudation of fumaric acid in CEA systems is a further avenue for studying the composition of CEA rhizospheres. Recent studies have indicated that azelaic acid, another organic acid, may act as a signaling molecule induced by microorganisms in the rhizosphere (Korenblum et al., 2020). The colonization of the rhizosphere by certain microorganisms can stimulate the accumulation of azelaic acid, where it will travel up the root system and induce a response known as the “Systemically Induced Root Exudation of Metabolites” or SIREM. Azelaic acid is one of the metabolites released during this SIREM response, where it aids in altering the rhizosphere against colonization by pathogens or undesirable microorganisms (Korenblum et al., 2020).

### 2.3 Sugars

Sugars exuded by vegetable crops in CEA systems can alter the composition of the rhizosphere (Lopes et al., 2022). They are a critical part of a substance developed in the rhizosphere known as “Mucilage.” Mucilage is a sticky substance comprised of exuded sugars which provides not only a suitable environment for soil dwelling microbes, but also a source of nutrients (Pettolino et al., 2012; Walker et al., 2003). As of recently, a majority of the studies involving exuded sugars have been restricted to large scale monoculture crops such as Maize or Soybean. Therefore, the functions and mechanisms involved with exuded sugars in CEA crops are speculative as of yet.

The most common sugars exuded by CEA crops are Xylose, Fructose, and Glucose (Kamilova et al., 2006). Xylose is a monosaccharide that is synthesized through the breakdown of hemicellulose—one of the components of plant cell walls (Harper and Bar-Peled, 2002). Xylose was found to be the primary sugar exuded by seeds and seedlings. The sugar composition of exudates changes with age (Vives-Peris et al., 2020). The sugar exudates of mature plants are composed of fructose and glucose, both monosaccharides, which often bond to each other to create sucrose. They are involved in a plethora of metabolic functions, perhaps most notably cellular respiration. Notably, there is a close connection between root morphology, phosphorus, and exuded sugars (Canarini et al., 2019). Compared to other nutrients, the Root Apical Meristem (RAM) is more sensitive to the abundance (or lack thereof) of phosphorus. When the levels of phosphorus at the root tip are detected as “low,” the plant first sends out organic acids, particularly citric acid, which activate a mechanism known as “RAM exhaustion.” RAM Exhaustion is a process by which the meristematic growth is halted, and sugars are sent to the root tip (Kamilova et al., 2006). When RAM Exhaustion begins, lateral root growth dominates the root architecture (See **Figure 1C**). While the exact mechanism is unknown, it can be supposed that the sugars sent to the RAM aid in the building of these lateral roots (Kamilova et al., 2006).

Maltose, though less commonly exuded, is a disaccharide which acts as a carbon and energy source for the microbe populations in the rhizosphere. The presence or absence of maltose in exudates can readily influence rhizosphere composition as well as rates of biological activity in this region (Wen et al., 2022). Maltose thereby acts as a primer of bioactivity in more developed traditional agroecosystems. Trehalose, another disaccharide, is one of the many components of stress mitigation response in plants. The exudation and subsequent reabsorption of trehalose acts as a signaling molecule which aids in osmotic protection during periods of drought stress (Eh et al., 2024). The accumulation of osmoprotectants, including trehalose itself, is a common strategy in the constant battle against abiotic stressors. Further investigation of these more complex sugars is necessary to better understand their presence and potential impact in CEA systems.

### 2.4 Microbial interactions

The microbial world of fungi, bacteria, algae, and various protists is nearly innumerable. Plants in common agroecosystems communicate via their roots with bacteria through the production of certain chemicals and the sensing of the response afterward (Ryan et al., 2016). This call and response can be initiated and well regulated by the plant via its root exudates (Bais et al., 2006). The root colonizing nutrient-fixing bacteria which live in nodules is perhaps one of the best-known plant-microbe interactions. It is a symbiotic relationship as well. To attract the rhizobacteria, legume species will secrete secondary metabolites known as flavonoids, signal to the bacteria, as well as triggers root system transformation—helping create the nodules which the rhizobia will inhabit (Micallef et al., 2009). The common species include *Bacillus*, and *Klebsiella*, whereas *Azospirillum* is a free-living soil-dwelling nitrogen fixer (Hnini and Aurag, 2024; Zhou et al., 2023; Abiraami et al., 2021). Nitrogen fixation is not the only skill of some of these rhizobacteria. Phosphate solubilizing bacteria, or PSB, species of *Pseudomonas, Rhizobium, Enterobacter*, are just a few of the species which can be signaled by plants under nutrient stress (Lei et al., 2023). Rock and mineralized phosphates are free to be broken down into simpler forms by these bacteria.

Such bacteria may also be involved in micronutrient acquisition as well. Nearly all the species of above mentioned bacteria secrete their own siderophores– small molecules designed to chelate iron from the atmosphere into available forms. Such siderophores produced by these beneficial bacteria often travel freely through the rhizosphere, improving the overall solubility of iron (Chiaranunt and White, 2024). Roots in close proximity to these siderophores can participate in uptake of this iron, supplementing a plant system’s existing iron accumulation methods (Abiraami et al., 2021). In CEA crops, plants have continued host populations of bacteria which produce siderophores despite ready access to micronutrients.

Though bacteria are the most efficient in this process, some fungi can be involved as well. *Aspergillus and Penicillium* are among these species. Rounding out the macronutrients, there are specialized exudations, namely gibberellins, abscisic acid, and ethylene which attract bacteria that break down Potassium (Meena et al., 2014). Again, the organic acids are the most common secretions which attract these (Xu et al., 2021). Potassium is typically bound up in rocks like muscovite, mica, and illite, which can be broken down potassium solubilizing bacteria (KSB). These usually aerobic species include *Pseudomonas, B. edaphicus*, and *B. mucilaginosus*. Much of these species have yet to be studied in CEA systems. Their potential impacts remain a topic of future study, with expected differences between soil based, and hydroponic based systems. The growing world of symbiotic microbe-plant interactions promises the possibility of exciting soil additives and soil fertility treatments, utilizing these unique properties in the future (Lei et al., 2023; Upadhyay et al., 2022.; Hu et al., 2018).

## 3 Changing environments, alters exudates

**Figure 2:**
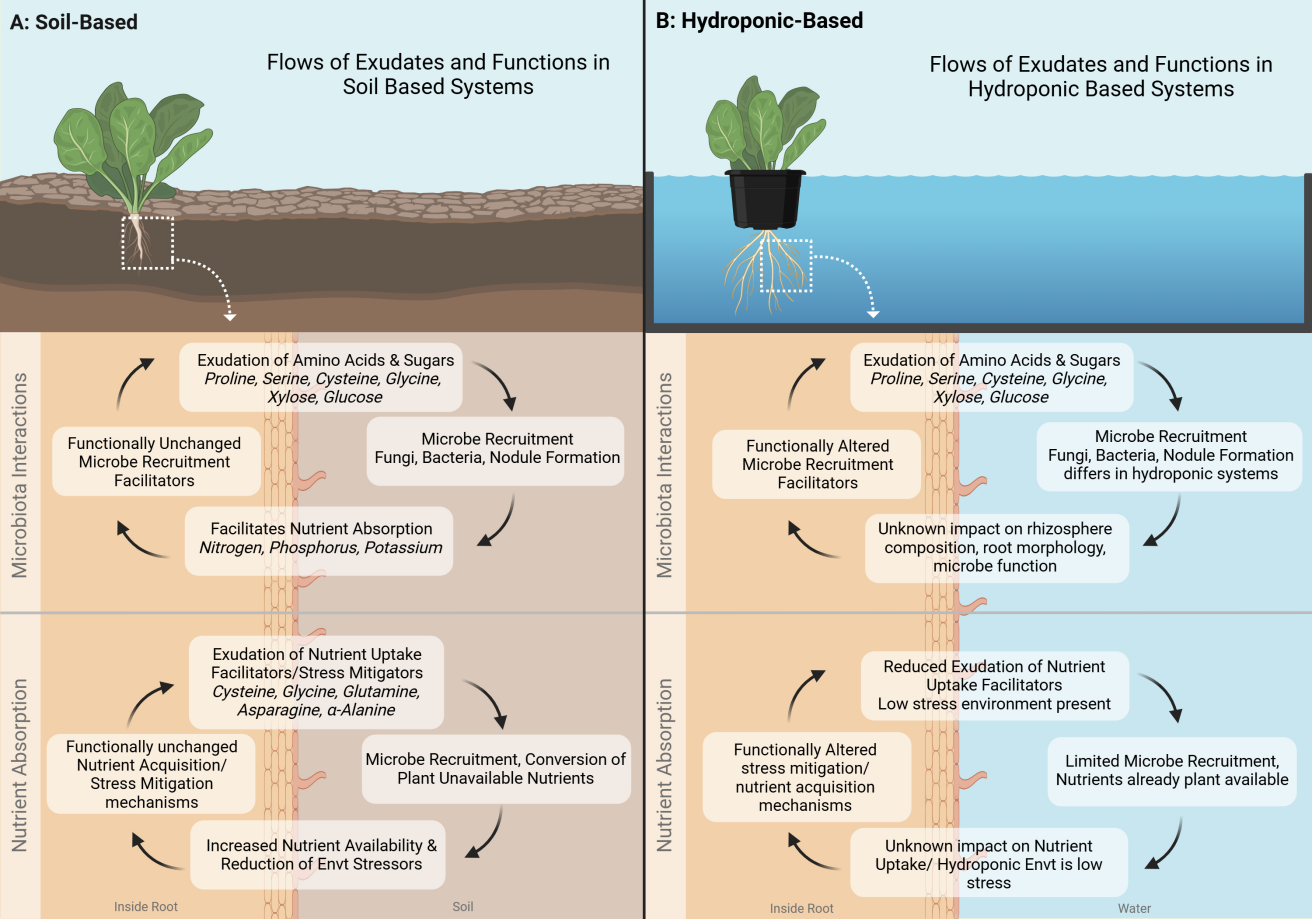
Comparison of Flows of root exudates and their functions in soil-based and hydroponic-based Controlled Environment Agriculture (CEA) systems. **(A)** Soil-Based System: Root exudates, such as amino acids (e.g., proline, serine, cysteine, glycine) and sugars (e.g., xylose, glucose), contribute to microbial recruitment, nutrient absorption, and stress mitigation. These functions remain largely unchanged in soil systems, where exudates facilitate interactions with fungi and bacteria, enhancing nutrient availability and environmental resilience. **(B)** Hydroponic-Based System: While root exudates in hydroponic systems contain similar compounds, their function is altered due to the absence of soil. Microbial recruitment is limited, and the impact of exudates on rhizosphere composition, root morphology, and nutrient uptake remains uncertain. Hydroponic systems exhibit reduced exudation of nutrient uptake facilitators due to the controlled, low-stress environment. Further research is needed to understand how these differences affect plant growth and system sustainability. Created in BioRender. Villouta, C. (2025) https://BioRender.com/u42f560.

Controlled environment agriculture can be conducted in a number of ways, with the primary distinction being soil-based systems vs water-based systems. Soil based systems include high tunnels, greenhouses, hoop houses, and growth chambers which utilize soil or soil substitute. Water-based systems are typically hydroponic systems such as ebb and flow, nutrient film technique, or deep water culture. These systems utilize an inert media such as rockwool, LECA clay pebbles, or styrofoam and supplement nutrition through fertigation. Differing systems lead to differing compositions of root exudates in controlled environment agriculture (see **Figure 2**) (Zhou et al., 2023). This is primarily due to four main factors including the substrate itself, biotic and abiotic stressors, nutrient availability, and microbial presence (Korenblum et al., 2020). Varying combinations of the aforementioned factors can lead to exudation that enhances plant growth or detracts from it.

Substrates play a key role in determining the composition of exudates by serving as a habitat for microorganisms as well as influence nutrient availability. Productive soil-based systems have a balance of high surface area, adequate water holding capacity, and high nutrient availability (see **Figure 2A**) (Hnini and Aurag, 2024). Exudates in these systems tend to be a blend of the aforementioned amino acids, organic acids, and sugars (Badri and Vivanco, 2009). The diverse blend of amino and organic acids can aid in nutrient uptake, while sugars can be utilized to attract beneficial bacteria (Dannehl et al., 2015). Water-based systems such as hydroponic systems, have inert media that have large surface areas for beneficial microorganisms. The media itself provides no nutrients as fertigation is typical. Water holding capacity is not of concerns due to a readily available water supply (see **Figure 2B**). In these systems, the substrate does not promote a balanced mixture of the three categories of exudates, instead it promotes those which can attract beneficial microorganisms (Hao et al., 2007; Kamilova et al., 2006).

Abiotic stressors are another differing factor which influences the composition of exudates in these systems (Šamec et al., 2021). Water-based systems can have salinity, pH, mineral content, and importantly water availability, regulated daily or even hourly depending on the sophistication of the equipment (Moncada et al., 2020; Vescio et al., 2022). Plants in soil based systems, which often cannot regulate these factors, must rely on exudates to aid them in their survival (Parthasarathy et al., 2019; Upadhyay et al., 2022). Thus under ideal conditions, the plants in the water-based system are likely to not exude amino acids which are a part of abiotic stress condition responses (**Figure 2B**).

Nutrient availability in soil systems is typically determined by the organic matter content, the soil structure, and the amount of fertilization. Some soil based systems such as coconut coir and perlite systems can sidestep this through fertigation, but the majority of outdoor CEA relies on the aforementioned factors to bring nutrients to the crops. Exudates in such systems include the “Nutrient Uptake Facilitator” branch of amino acids including Asparagine, Cysteine, Glycine and α-Alanine (Trovato et al., 2021). Balanced water-based systems are less likely to have high concentrations of these exudates as their nutrient availability is high from consistent fertigation efforts (Lei et al., 2022).

Microorganisms are a driving factor in the differentiation of exudates in soil vs water-based systems. Studies delving into the microbial populations that exist in water-based CEA systems are limited. These systems can be sterile or nonsterile, and utilize substrates which may create surface area for microbiota or not (Mavrodi et al., 2021). Section three will explore a case study from the limited literature regarding hydroponic microorganisms. The literature of soil-based systems has broadened significantly since the discovery of nitrifying root-colonizing bacteria (Canarini et al., 2019). Along with rhizobacteria, various beneficial fungi have been identified as well (Bais et al., 2006). As explained in section one, plants exude sugars in order to attract these bacteria (Korenblum et al., 2020; Martinez-Hidalgo and Hirsch, 2017). In water-based systems, these sugars may simply flow into a sump tank and bypass the ability to attract microorganisms completely (**Figure 2**). Despite this, the literature reveals that even in higher flow hydroponic systems, plants will continue to exude sugars (Ryan et al., 2016).

## 4 Case studies

Having introduced a theoretical perspective on the common compounds exuded in vegetable and fruit crops, it may be helpful to delve into case studies to better understand specific impacts and implications of certain exudates and controlled environmental conditions. This section will explore four case studies which introduce differing environmental conditions and explores their impacts on exudate secretion, makeup, and plant impact. For a complete list of case studies referenced below, as well as others pertinent to CEA systems see **Table 2**.

**Table 2:** Guide to case study literature on CEA exudates.

System	Crop	Exudate	Citation
Hydroponics	Cabbage + Lettuce	Organic Acids	Kasukawa and Miyazawa (2020)
Hydroponics	Leafy Greens	Citric Acid	Miyazawa, 2016
Hydroponics	Tomato, Cucumber, Pepper	Glucose, Fructose, Citric Acid	Kamilova et al., 2006
Hydroponics	Spinach	Oxalate	Yang et al., 2005
Hydroponics	Watermelon	Allelochemicals	Hao et al., 2007
Hydroponics	Watermelon/Rice	Allelochemicals, Organic Acids	Hao et al., 2010
Greenhouse	Tomato	Phenolics	Yang et al., 2016
Hydroponics	Tomato, Red Pepper, Cucumber	Amino Acids	Vančura and Hovadík, 1965
Perlite	Watermelon	Malic & Citric Acid	Ling et al., 2011
Vermiculite	Strawberry	Phenolic Acids	Cao et al., 2016
Hydroponics	Lettuce	Organic Acids	Lee et al., 2006
Hydroponics	Pepper, Lettuce	Allelochemicals	He et al., 2012
Soil	Pepper	Amino, Organic Acids	Marschner et al., 1997
Hydroponics	Cucumber	Allelochemicals, Organic Acids	Pramanik et al., 2000
Soil	Lettuce	Amino Acids, Sugars	Neumann et al., 2014
Soil	Lettuce, Cabbage	Organic Acids	Dhungana et al., 2023

### 4.1 Organic acid content in water stress conditions

In controlled environment production, seedlings are grown separately from the main production area and are transplanted into the main growth and finishing area when they are still small. During this process, seedlings often become stressed and can be damaged (Kim et al., 2018). To alleviate this, it has been proposed to dose seedlings with a treatment of organic acids. Previous studies (Miyazawa, 2016; Kasukawa and Miyazawa, 2020) have established that treatments of organic acids, particularly citric acid, were effective in alleviating drought stress in leafy vegetables. Miyazawa (2016) proposed that Citric acid uptake through the roots acted as a signaling mechanism to leafy vegetables to increase antioxidant enzymatic activity. In turn, this should reduce oxidative damage stemming from water-related stress. To measure the potential damage mitigation effects of dosing seedlings, Kasukawa and Miyazawa (2020) grew cabbage and lettuce seedlings in hydroponics and subjected them to simulated drought stress. A measurement of drought stress response– oxidative enzymatic activity– was conducted focusing on the catalase and ascorbate peroxidase enzymes. Samples that were subjected to drought conditions had increased concentrations of various organic acids, including Citric, Tartaric, Lactic, and Aspartic acid. The presence of several organic acids exuded prompted the authors to conclude there was not sufficient evidence to state that organic acids absorbed through the roots could act as signaling molecules which reduced oxidative stress. Notably, this study was conducted in a hydroponics system. Studies done in soil-based production systems have found either differing results, or results which reveal a possible interaction between the roots, exudates, and soil microbiome (Gargallo-Garriga et al., 2018). Kasukawa and Miyazawa (2020) note this before going on to explain that there may be a role for exogenous applications, particularly foliar applications, of organic acids to reduce drought stress. There is certainly room for further analysis in this area, specifically in hybrid systems with microbial friendly hydroponic media where results may be different.

### 4.2 Bacterial biostimulants

Organic acids are not the only way to alleviate abiotic stressors (Moncada et al., 2020; Vetrano et al., 2020). There are many CEA sites worldwide which have elevated salinity concentrations in their groundwater. Cultivar breeding for salinity tolerance has only been able to aid in a limited fashion. Previous studies have indicated that populations of microorganisms may help salinity tolerance. Moncada et al. (2020) aimed to understand the impact of using beneficial bacteria as a biostimulant in hydroponic systems. Lettuce was grown in a hydroponic float based system in both fall and spring with four differing conditions based on a factorial combination. Two systems had a bacteria biostimulant solution containing the plant growth promoting *Bacillus amyloliquefaciens*, *B. brevis*, *B. circulans*, *B. coagulans*, *B. firmus*, *B. halodenitrificans*, *B. laterosporus*, *B. licheniformis*, *B. megaterium*, *B. mycoides*, *B. pasteurii*, and *B. subtilis* to their nutrient solutions while two systems acted as a control. Two systems were dosed with a 20mM solution of NaCl. The system without bacteria which was subjected to salt stress showed significant loss of yield and reduced growth, in some cases with a shoot mass reduction up to 60%. Whereas the system with bacteria which was subjected to salt stress was able to minimize reduction in growth, returning it to near salt free levels. Even in salt-free systems with the biostimulant added, fresh shoot mass was elevated compared to the control. Furthermore, the spring season showed the greatest impact of the biostimulant solution. Hence, the bacterial biostimulant was able to successfully mitigate the stress caused by increased salinity and increase the overall salt tolerance of lettuce, thereby demonstrating the capacity for plant growth promoting bacteria to alleviate abiotic stress (Moncada et al., 2020).

### 4.3 Microbiota friendly substrates

As noted in the previous section, studies done under soil conditions appear to have differing results from inert, inorganic, neutral media. In hydroponics, this often means water, styrofoam, or glass beads as substrates. In an effort to create a hybridized system with a media that can be colonized by beneficial microbiota, researchers have used spun-rock: often characterized as rockwool or stonewool and commonly used in hydroponic and aquaponic systems (Dannehl et al., 2015). Spun-rock media, though still inert and neutral, has increased surface area and dry areas which can allow various species of bacteria to colonize and exhibit beneficial effects normally observed in soil (Kamilova et al., 2006). In a joint Dutch/Russian study, stonewool (Spun-rock) substrate was used in a hydroponic system where tomato, cucumber, and sweet peppers were grown. Their plants exuded the major organic acids, citric and succinic acid, along with major sugars glucose and fructose (Kamilova et al., 2006). These exudates prompted microbial colonization substantiated through the detection of increased concentrations of advantageous “root-tip-colonizing” genera *Pseudomonas* (Kamilova et al., 2006). These bacteria rely on the organic acid exudates, primarily citric acid, as a carbon source and in turn synthesize plant growth signaling molecules, notably auxin precursors, which help to stimulate plant growth (see **Figure 1B**). This effect was particularly noted in the two fruit crops: tomatoes and sweet peppers. While other organic acids varied in their concentration depending on plant age, citric acid content stayed relatively high for all ages of the plant. Malic acid, which is important in juvenile stages for promoting the production of chlorophyll in smaller plants, decreased as the plants reached more mature stages. The opposite was true with regards to succinic acid– an important part of the citric acid cycle. In soil, where results of hydroponic studies have been reaffirmed, succinic acid has also been shown to be secreted by the roots in response to colonization by root-tip and rhizospheric bacteria. The next section will examine how these bacteria and secondary metabolites can contribute to disease control, particularly in fruit crops.

### 4.4 Countering contamination

Aluminum contamination has been a noted concern in Controlled Environment Production in urban areas. Sites with soil that has been previously contaminated due to waste production runoff, spillage, or other contaminating events may pose an issue to not only food safety but also plant production. However, some plants have been known to develop Aluminum mitigation responses, particularly in their underground root structures (Ofoe et al., 2023). One such response is the efflux of various organic acids (see **Figure 1D**) (Chandra and Keshavkant, 2021). Yang et al. (2005), measured the efflux rate of organic acids of Spinach (*Spinacia oleracea*) under exposure to aluminum to identify a detection and response mechanism. Young spinach plants grown under hydroponic conditions were exposed to a solution of 0.5 mM CaCl_2_ containing varying concentrations of Aluminum Chloride. After six hours of exposure, the exudates were collected and analyzed. The organic acid Oxalate was noted to efflux constantly for six hours, but increased in concert with greater Aluminum concentrations. In the root tip, where aluminum can more easily penetrate, the efflux was 5x that of the rest of the root system. To further investigate whether this response was Aluminum-specific, the researchers attempted to trigger the Oxalate efflux utilizing phosphorus, and with a cation lanthanum. Results demonstrated Oxalate efflux was not affected by phosphorus indicating that spinach has aluminum-specific resistance mechanisms (Yang et al., 2005). The understanding and application of such mechanisms is critical for urban CEA production in soils which may have mild aluminum contamination, or areas which may be growing produce that is not suitable for human consumption (Chandra and Keshavkant, 2021). As CEA expands into (peri)urban landscapes, finding plants with mechanisms that can make them suitable for production in non-optimal areas is a key challenge that must be addressed.

Other novel studies have established several non-CEA crops as potential bioremediation agents which could work in tandem with CEA crops on contaminated sites. Petroleum hydrocarbons remain a key source of contamination among urban landscapes and pose a challenge for production in said sites. To measure the potential viability for utilizing a non-CEA crop for use in a hydrocarbon contaminated site, Eze and Amuji (2024) grew Alfala (*M. sativa*) in pots with 300 grams of petrodiesel mixed in. After 90 days, the plants were removed from pots and soil was sampled from 3 locations: roots, 1 cm from roots, and 3 cm from roots to measure both exudate content and petrodiesel contamination. Acetic, Malic, and Oxalic acid were noted organic acid exudates, along with fructose and glucose as sugars, and Lysine as the primary amino acid exuded. Phytoremediation by the plants was significant in dissipating the diesel fuel hydrocarbons from the contaminated soil. Furthermore, distance from the root is a factor in the biodegradation of said hydrocarbons, with the closest total amount of degradation in soil attached to the root (Eze and Amuji, 2024). Therefore, exudates can aid in the bioremediation of other contaminants in urban environments. The exudates provide optimal environments to bacteria and microorganisms which can degrade the present hydrocarbons and/or catalyze them into other less harmful compounds.

### 4.5 Allelopathic potential of root exudates

Allelopathic compounds constitute a multitude of low-weight molecular compounds which are exuded from plants during various stages of secondary metabolism (Hao et al., 2007). First described in the 1930’s, they range in toxicity, concentration, effect, and composition (Cheng and Xu, 2013). As of late, they have begun to be studied as a part of monocropping systems due to their ability to suppress diseases that typically invade continuous cropping systems (Sheldon et al., 2021; Cheema et al., 2004). With the increasing role of controlled environment agriculture, allelopathic potential has become a prescient issue, particularly among hydroponically grown fruit and vegetable crops. Studies conducted by Hao et al. (2010; 2007) measured the impacts the allelopathic exudates produced by mature hydroponically grown watermelon on watermelon and lettuce seedlings. Watermelon can be prone to autotoxicity, and disease susceptibility– particularly in continuously cropped systems. Utilizing a continuous root exudate tapping system (see **Figure 2** CRETS, Hao et al., 2007) the researchers were able to measure the exuded allelopathic compounds over the course of one-hundred days. Results indicated that all tissues in the mature watermelon plants contained and exuded allelopathic compounds, and that these compounds had inhibitory effects particularly on the radicle elongation of watermelon and lettuce seedlings (Hao et al., 2007). However, as this study only observed the growth of other plants, it could not conclude on the potential for allelochemicals to mitigate crop diseases. For that, we turn to our next case, which measured the capabilities for allelochemicals to combat common watermelon diseases.

### 4.6 Allelopathic disease mitigation

Fusarium wilt is a common disease in watermelon crops caused by the fungi *Fusarium oxysporum*. It is a pathogen which decimates watermelon yields, particularly in continuous cropping systems (Rahman et al., 2021). Early research indicates an interaction between root exudates of watermelon and *Fusarium*. To qualify this interaction, and compare it to known allelopathic compounds, Hao et al. (2010) grew watermelon and rice hydroponically and measured the impact of their exudates on spore germination and sporulation of *Fusarium*. Rice has been previously demonstrated to have inhibitory effects on the growth of *Fusarium* spore germination and sporulation. Watermelon root exudates were found to significantly increase the growth of *Fusarium* while rice crops had anti-fungal properties which significantly limited the growth of the fungus. Thus, an intercropping system of rice with watermelon could successfully mitigate fungal growth in hydroponic systems and in turn reduce disease pressure, leading to increased watermelon yields (Hao et al., 2010). This research demonstrates that the disease mitigation properties of root exudates differ from crop to crop– particularly among fruit crops as will be discussed in the next example.

Pest mitigation responses differ significantly between CEA crop species– especially those that involve fruit development. Tomato crops are susceptible to a number of pests, with nematodes being a consistent source of pest pressure (Pulavarty et al., 2020). Yang et al. (2016) grew tomato crops in a greenhouse and inoculated the roots with *Meloidogyne incognita*, a common nematode to tomato production. The tomato root exudates were found to suppress the egg hatching capabilities of *M. incognita*. Furthermore, exudates increased the mortality of juvenile *M. incognita* hatchlings. In response to inoculation, phenolic compounds, known to have allelopathic effects, increased significantly. The set of secreted compounds appeared to have three functions. Dibutyl phthalate served to repel *M. incognita*, thereby preventing eggs from being laid. 2,6-Di-tert-butyl-p-cresol and dimethyl phthalate suppressed eggs from hatching. L-ascorbyl 2,6-dipalmate was correlated with increased juvenile mortality once the eggs hatched. The four compounds worked together to mitigate nematodal infection (Yang et al., 2016). The researchers did note that other studies show that certain compounds work to attract nematodes and other pests rather than repel them. Based upon prior literature, it can be postulated that these mechanisms have most likely been developed to attract beneficial microbiota such as rhizobia, but have the inadvertent effect of attracting certain pests instead (Cao et al., 2016).

CEA crop species need not work alone. A study by Zhou et al. (2023) incorporated a soil-based Tomato CEA system with a potatoonion (*Allium cepa*) system. This intercropped system aimed to study how microbial recruitment by a neighbor plant could impact disease mitigation in the target plant. Treatments consisting of an intercropped system and a monoculture tomato system were grown for four weeks before being challenged by *Vertticillium dahliae* or Verticillium wilt, a common fungal pest in tomato monoculture. After two weeks, the severity of the infections were quantified. The results indicated that the intercropping system altered the microorganism composition of the tomato rhizosphere. *Bacillus*, a common beneficial bacteria in agroecosystems as established in prior sections, was more abundant in tomato rhizospheres which were intercropped. In turn, this increased the disease suppression capabilities of the tomato system. The presence of *Bacillus* has negative impacts on fungal mycelium growth of Verticillium wilt, thereby inhibiting its growth and spread (Zhou et al., 2023). The capacity for disease mitigation via allelopathic intercropping has yet to be studied in depth. Thus, it becomes clear that the allelopathic effects of root exudates are only beginning to be understood, and have the potential to change cropping systems as researchers develop a better understanding of the network and cross-talk of root exudates and allelochemicals.

## 5 Discussion

While much headway has been made over the past two decades in understanding the composition and role of root exudates in controlled production systems, there are still several avenues of further exploration in CEA root exudates. For many years, root exudate research was focused on industrially produced field crop species such as maize, wheat, and soybean. From there, a robust database of exudates, their functions, and possible endogenous or exogenous applications was created (Zhou et al., 2023; Canarini et al., 2019). As research began to branch out in the early 2010’s, studies continued to be focused on select crops expanding to include alfalfa and cover crops (Eze and Amuji, 2024). The limited number of studies outside of a few select CEA crops, namely tomato, lettuce, and pepper, along with an indoor production sector wide focus on strictly hydroponic production has left a sizable knowledge gap to be filled. Notably, studies regarding production in large scale fields are few and far between. An exhaustive search of the literature revealed just under twenty studies (see **Table 2**) with direct implications in CEA root exudate crop production. Aforementioned studies in ecological niches or less cultivated species could create a database connecting crop species to specific exudates, and their applications. Such databases would be beneficial to the burgeoning CEA industry and urban agriculture development. In conjunction, the exogenous application of specific exudates, such as organic acids or sugars has yet to be studied in depth. Previously highlighted research has demonstrated the capability for such applications to aid in several different areas. Stress resilience and response is among the most coveted of such applications, with studies by Dhungana et al. (2023) and Kasukawa and Miyazawa (2020) demonstrating the capacity to alleviate such abiotic stressors. The exact dosages of such applications, if provided exogenously, have yet to be determined but remain an avenue of exploration in CEA systems. Further alleviation of stress by exudate scavenging of ROS, specifically under high heat or drought conditions remain unknown. Of interest are the mechanisms and pathways by which such responses occur and begin to mitigate such threats (Bais et al., 2006). While the potential for nutrient acquisition through or in conjunction with exudates has begun to be explored, the efficacy and potential supplementation of nutrition has yet to be determined (Lei et al., 2022; Trovato et al., 2021). That is, how much could exudation or exogenous application of exudates increase the amount of available nutrients in CEA systems? Hydroponic-based systems with consistent fertigation would not benefit in such a scenario but soil-based or technology-limited CEA applications in field conditions could gain significant insight.

Shifting to a perspective on microorganisms and the rhizosphere in relation to other plants reveals several areas of interest. Foremost, recent studies have begun to qualify and quantify the multitude of microorganisms in CEA systems (Vives-Peris et al., 2020). Soil-based systems, with a more established database of such organisms have turned their attention to interactions between plant, soil, and microorganisms. However, such an approach remains in its infancy for hydroponic based systems. Though, the literature has established that such communities can and do exist in such systems as well (Canarini et al., 2019). A qualification of such systems could inform the greater agricultural community of the role of such microorganisms in a multitude of pathogen protective and allelopathic capacities. Specifically, there is a novel area involving the impacts of exudates and microbial recruitment on neighboring plants and their root systems which remains relatively unexplored. Tritrophic interactions– that is the interactions between plant, microbe, and nematode have yet to be investigated as CEA systems strive to be more sterile ecosystems (Badri and Vivanco, 2009 A database of microorganisms, along with their allelopathic disease prevention or pathogen protection in common CEA crops such as spinach, lettuce, or tomato would be of high value (Chiaranunt and White, 2024).

The potential for contamination cleanup in soil-based systems established by Eze and Amuji (2024) has continued the well-investigated trend of using non-CEA crops for bioremediation. There is a clear gap, along with a motivation for the usage of CEA crops for the same purpose in urban landscapes or vertical farming (Chiaranunt and White, 2024; Mavrodi et al., 2021; Badri and Vivanco, 2009). The mechanisms of “countering contamination” have been established by Yang et al. (2005) but the full breadth of contaminants, as well as the efficacy and long-term impacts of CEA bioremediation must be further explored. Intercropping to aid in such bioremediation efforts, or the effects of exogenous application of specific exudates, namely organic acids or sugars to stimulate microbial colonization of the rhizosphere remain unknown. Though intercropping has been established as aiding in defense of target crops in CEA systems, the mechanisms by which it may or not aid in contaminant mitigation should be a target of future studies. Notably, the case studies in section three demonstrate the myriad of capabilities along with the “proof of concept” that there is agronomic potential and significant relevance to CEA research (Zhou et al., 2023; Eh et al., 2024).

One final note is that as climate change continues to alter environmental conditions, it would be prudent to study alterations in CEA crop exudates under anticipated conditions including high heat, increased carbon dioxide concentrations (ranging from 400-1200ppm) and limited water availability– perhaps as low as 50% of current usage. With a world population projected to continue growing at a rapid pace, along with the industrialization of several major countries in the global south, global CEA production will continue to grow as well. More efficient utilization of resources can be achieved through expanding our database of root exudates, their functions, pathways, and impacts on plants and production systems.
